# Perovskite light-emitting/detecting bifunctional fibres for wearable LiFi communication

**DOI:** 10.1038/s41377-020-00402-8

**Published:** 2020-09-16

**Authors:** Qingsong Shan, Changting Wei, Yan Jiang, Jizhong Song, Yousheng Zou, Leimeng Xu, Tao Fang, Tiantian Wang, Yuhui Dong, Jiaxin Liu, Boning Han, Fengjuan Zhang, Jiawei Chen, Yongjin Wang, Haibo Zeng

**Affiliations:** 1 MIIT Key Laboratory of Advanced Display Materials and Devices, Institute of Optoelectronics & Nanomaterials, School of Materials Science and Engineering, Nanjing University of Science and Technology, Nanjing, 210094 China; 2 Peter Grünberg Research Center, Laboratory of Broadband Wireless Communication and Sensor Network Technology, Ministry of Education, Nanjing University of Posts and Telecommunications, Nanjing, 210003 China

**Keywords:** Inorganic LEDs, Optical sensors, Nanoparticles, Optoelectronic devices and components, Displays

## Abstract

Light fidelity (LiFi), which is emerging as a compelling technology paradigm shifting the common means of high-capacity wireless communication technologies, requires wearable and full-duplex compact design because of its great significance in smart wearables as well as the ‘Internet of Things’. However, the construction of the key component of wearable full-duplex LiFi, light-emitting/detecting bifunctional fibres, is still challenging because of the conflicting process between carrier separation and recombination, as well as the highly dynamic film-forming process. Here, we demonstrate light-emitting/detecting bifunctional fibres enabled by perovskite QDs with hybrid components. The hybrid perovskite inks endow fibres with super-smooth QD films. This, combined with the small exciton binding energy and high carrier mobility of perovskite QDs, enables successful integration of electroluminescence and photodetection into monofilaments. The bifunctional fibres possess the narrowest electroluminescence full width at half maximum of ~19 nm and, more importantly, the capability for simultaneously transmitting and receiving information. The successful fabrication of narrow emission full-duplex LiFi fibres paves the way for the fabrication and integration of low crosstalk interoperable smart wearables.

## LiFi technology: Hybrid fibres for bidirectional communication with light

Light-emitting and detecting fibres incorporating ‘quantum dot’ structures composed of material called perovskite suitable for wireless communication using light, known as light fidelity (LiFi). The fibres, which should be suitable for developing wearable smart devices, have been developed by Qingsong Shan and colleagues at Nanjing University of Science and Technology, China, with co-workers at Nanjing University of Posts and Telecommunications, China. Quantum dots are tiny nanoparticles that are sufficiently small for quantum mechanical effects to permeate across their entire structure. Perovskites share the crystal structure of the natural mineral perovskite, with varying chemical compositions that open up a wide range of new properties. The perovskite quantum dots are coated as an active layer on polyethylene terephthalate (PET) layers, creating a fibrous device with the much sought property of simultaneously transmitting and receiving information.

## Introduction

A vast amount of data is generated every day in this information era, which has triggered the development of new high-speed wireless data communication technologies, such as the fifth generation of cellular systems (5 G) and light fidelity (LiFi). This, combined with the emerging fields of the ‘Internet of Things’ (IoT) and big data, persistently pushes the miniaturization and densification of wireless communication terminals. In this context, the targeted design of wearable LiFi with the merits of portability, conformability, and safe and high-speed wireless communication will have boundless prospects in IoT terminals. Nevertheless, a typical LiFi system always consists of considerable quantities of transmitters (LEDs) and receivers (PDs) to meet the demand for high-capacity wireless communication and multiuser interaction. This definitely increases the complexity and volume of LiFi equipment, hindering the construction of wearable LiFi. The construction of full-duplex (bilateral real-time communication capable) wearable LiFi will be helpful for obtaining higher data rates and more portable IoT terminals^1^.

Currently, rapid advances in material science and processing technology have made versatile wearables with functions such as health care, data transfer, energy storage, and military and fashion applications possible^2–9^. Therein, fibres have been proven to be excellent platforms for the construction of versatile functional devices^9–17^ as well as carriers of receiver and emitter diodes in wearable optical communication systems^4^. In terms of the data transmission part in wearable LiFi, electroluminescent (EL) fibres based on inorganic phosphors^18^, organic materials^15,19^, and polymers^9,20^ have already been reported, but they all have some shortcomings and are not suitable for the construction of wearable LiFi. For example, EL fibres based on inorganic phosphors require an alternating current (AC) field to create polarization currents to realise electroluminescence, and they suffer from inhomogeneous emission and a high operating voltage, which prevent them from being safely and conveniently integrated into textiles. Polymer light-emitting electrochemical cells usually have a slow turn-on process because of the p-i-n junction formation requiring time for the diffusion of mobile ions, which is not suitable for data transmission. The widely used thermal evaporation process in the fabrication of OLED fibres is costly. More importantly, a broad EL full width at half maximum (FWHM) is ubiquitous in the above devices, and the overlap of EL spectra between different EL devices will certainly bring about channel crosstalk in the operation of the LiFi system based on wavelength-division multiplexing technology.

Apart from the demands for high luminescence quality and an inexpensive fabrication process, integrating the detection function into a single EL fibre is also necessary for wearable LiFi. Although light-emitting/detecting bifunctional devices have been realised on rigid substrates, including in double-heterojunction CdS/CdSe/ZnSe nanorod^21^, bilayer MoTe_2_ p–n junction^22^, and InGaN/Al_0.10_Ga_0.90_N multiple-quantum-well^23^ based devices, these device structures and materials are not suitable for wearable light-emitting/detecting bifunctional fibres. The construction of such bifunctional devices is still challenging because of the conflicting process between carrier separation and recombination as well as the highly dynamic film-forming process^24^; suitable materials and device structure are needed. Quantum dots (QDs), particularly metal halide perovskite QDs, have been extensively used for the construction of optoelectronic devices due to their excellent photoelectric properties^25–30^. Considering the easily photogenerated charges of perovskite QDs and their easily adjustable ink components^31^, the challenge of the conflicting process between carrier separation and recombination, as well as the highly dynamic film-forming process for constructing light-emitting/detecting bifunctional fibres, can be handled.

Here, we demonstrate a perovskite QD-based light-emitting/detecting bifunctional fibre obtained through a hybrid strategy and a facile, reproduceable solution assembly process. The hybrid perovskite inks improved the film-forming process, resulting in super-smooth QD films as well as successful fabrication of electroluminescent fibres. The green-emitting perovskite fibre has an FWHM of ~19 nm, which is the narrowest electroluminescence linewidth among all electroluminescent fibres. The emitting perovskite fibre can simultaneously receive information due to the small exciton binding energy and high carrier mobility of the perovskite QDs. The successful fabrication of narrow emission full-duplex LiFi fibres paves the way for the fabrication and integration of low crosstalk interoperable smart wearables.

## Results

### Fabrication of perovskite fibres

**Fig. 1:**
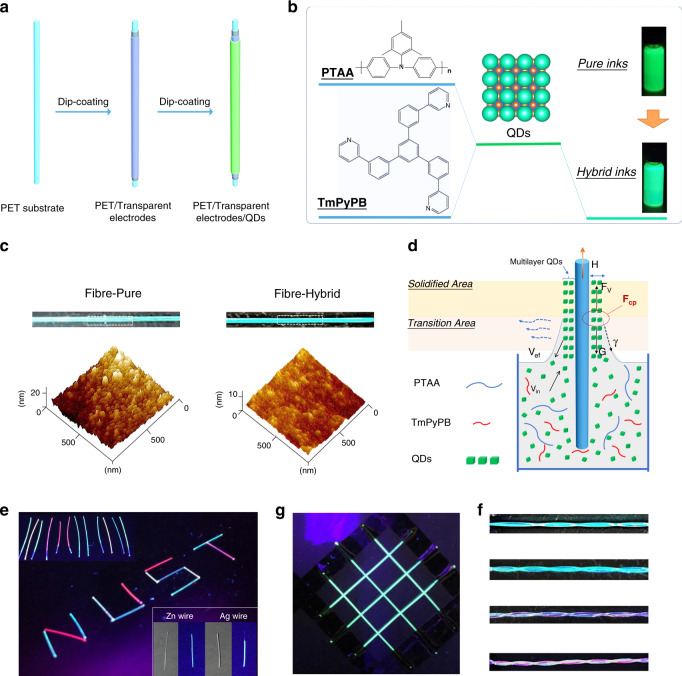
Fabrication of LED-photodetector bifunctional multilayer perovskite fibres. **a** Schematic illustration of the multiple dip-coating process of transparent electrodes and QDs in the fabrication of a perovskite fibre. **b** Schematic of the hybrid strategy of QD inks. **c** Photographs of as-prepared QD fibres, and corresponding AFM images. **d** Schematic illustration of the dynamic dip-coating process. **e** Perovskite fibres arranged into a ‘NUST’ pattern under UV illumination (RGB emission fibres were made with hybrid CsPbI_3_, CsPbBr_3_, and CsPb(Br/Cl)_3_ inks). Insert, perovskite fibres being prepared on Zn and Ag wires, and their corresponding PL images under UV illumination. **f** Photographs of the perovskite fibres woven together into a lattice under UV illumination. **g** Photograph of perovskite fibres assembled into one multicolour emission fibre under UV illumination.

The coaxial quantum dot light-emitting fibre (QLEF) was fabricated layer by layer through a typical dip-coating process. As shown in Fig. 1a, a transparent PET fibre was used as the substrate for the construction of the coaxial structure flexible QLEF. The transparent electrodes and QD layer were subsequently dip-coated on the 0.3 μm diameter polyethylene terephthalate (PET) fibre. It is well known that both appropriate viscosity and surface tension of the solution are crucial for the construction of a high-quality electroluminescent (EL) layer in the dip-coating process. Generally, it is difficult to dip-coat homogeneous and smooth perovskite QD films via the regulation of the solvent and concentration. It is difficult to control the QD film-forming stage in the dip-coating process because the surface tension of QD ink is rather low, which usually makes QDs spread easily^24^. For a single-step dip-coating process, QDs typically form a network structure decorated by isolated islands of aggregated QDs (Supplementary Fig. S1). For a multiple-step dip-coating process, the QD film usually has a large surface roughness. The heterogeneous structure will create a short-circuit current and joule heating under an electric field when applied as a luminous layer of EL devices, which will seriously deteriorate the luminance uniformity of EL fibres or even disable the EL fibre device. Herein, we propose a hybrid QD ink system, which consists of poly[bis(4-phenyl)(2,5,6-trimethylphenyl)amine poly(triarylamine) (PTAA), 1,3,5-tri[(3-pyridyl)-phen-3-yl]benzene (TmPyPB), and perovskite QDs (Fig. 1b). The green emission of pure QD ink changes to cyan after the addition of PTAA and TmPyPB under UV irradiation. Meanwhile, the emission peak of the perovskite QDs is slightly blue shifted (Supplementary Fig. S2), which is probably because of the change in the average size of the self-assembled QDs in the organic matrix^32^.

Figure 1c demonstrates AFM images of dip-coated films obtained by using the pure QD and hybrid QD inks under the same procedure. The as-prepared hybrid QD film exhibits a super-smooth surface with a roughness of 1.9 nm, while the film dip-coated with pure QDs has a rather rough surface with a roughness greater than 10 nm. This is further confirmed by the PL mapping results (Supplementary Fig. S3). The fused NCs in the hybrid film and the possible coupling between QDs may also help improve the luminescent properties with the assistance of multiexciton resonances^33,34^. We believe that the successful fabrication of this super-smooth film is probably due to the improved film-forming process of the hybrid QD ink. Figure 1d illustrates a typical dip-coating process. In brief, the fibre is first dipped into and wetted by the QD ink, followed by the vertical withdrawal process. In the film-forming process in dip-coating, the film thickness is controlled by the coefficient of the gravity force (G), viscous drag (F_v_) and liquid-vapour surface tension (γ)^24,35^. Typically, the dip-coating process can be divided into the transition stage and solidified stage. Draining and evaporation of the solvent accompany the balance between the QD efflux and influx below the liquid−gas−solid three-phase contact line in the transition stage. Usually, solvent evaporation often results in many pores in the compliant film. The pore-containing structure collapses in the solidified stage due to the effect of the capillary force (F_cp_), which is proportional to the surface tension and viscosity of the ink. Inevitably, the viscosity and surface tension of hybrid QD inks are increased after the addition of organic components (Supplementary Fig. S4 and Table 1), which creates a greater F_cp_, thereby forming a super-smooth film through a cooperative effect of the gravity force, viscous drag and liquid-vapour surface tension. In addition, the gap-filling effect of small molecules and polymers in QD inks also contributes to a super-smooth film^38^. In contrast, the rough film dip-coated using pure QD inks was probably caused by aggregation of QDs, thermal expansion and solvent evaporation during the multiple dip-coating and thermal treatment processes. As a result, dip-coated fibres with homogeneous photoluminescence can be obtained. As shown in Fig. 1e, the perovskite fibres can be arranged into an ‘NUST’ pattern and exhibit homogeneous PL properties. In addition, such a composite strategy can be extended to various kinds of substrates, such as Ag wire and Zn wire. The bright and uniform photoluminescence under UV light is shown in the insert of Fig. 1e. The photoluminescent perovskite fibres can also be assembled into luminous textiles (Fig. 1f) as well as one fibre to achieve multicolour woven structures (Fig. 1g), showing great potential for the fabrication of perovskite EL fibres.

**Table 1:** Comparison of the FWHM of reported fibre-shaped electroluminescent devices.

Device Type	Emitting materials	FWHM (nm)	Peak position (nm)	Cation
OLED	Alq_3_/super yellow	113/67	518/531	^36^
OLED	Super Yellow	93	545	^15^
OLED	Alq_3_	~86	536	^19^
OLED	Super Yellow	~86	550	^37^
PLEC	PF-B/ETT-15/LiTf	~100	496–550	^9^
ACPEL	ZnS:Cu, Cl	85	493	^18^
Our work	Perovskite QDs	~19	~516	–

## Discussion

### Electroluminescence properties of the perovskite fibres

**Fig. 2:**
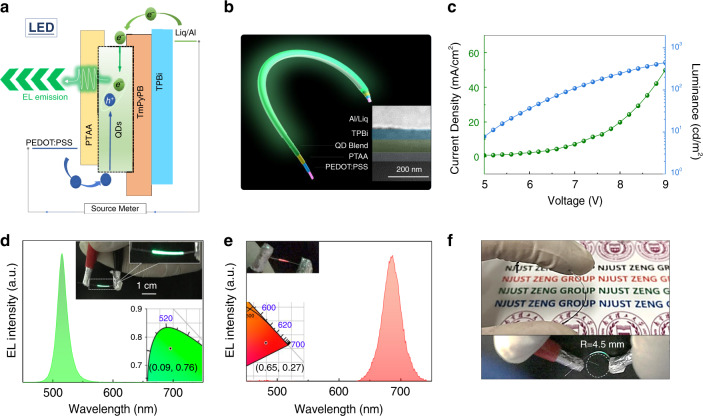
Electroluminescence function of the perovskite fibres. **a** Schematic illustration of the working mechanism (LED mode) of the light-emitting/detecting bifunctional perovskite fibre. **b** Schematic of the structure of a perovskite EL fibre. Insert, cross-sectional SEM image of a perovskite fibre. **c** Current density-voltage-luminance characteristics of an electroluminescent perovskite fibre. **d, e** Electroluminescence spectra of a green and a red electroluminescent perovskite fibre (inset schematics: photograph of the green and red electroluminescent perovskite fibres; chromaticity coordinates of the green and red electroluminescent perovskite fibres). **f** Photographs of a bent perovskite fibre and a bent fibre in operation.

As shown in Fig. 2a, the device structure of EL fibres was designed according to the typical planar device structure. PTAA, TPBi, and Liq/Al were selected as the hole transport layer (HTL), electron transport layer (ETL), and cathode, respectively. PEDOT:PSS (Clevios PH1000) was used as a high conductivity transparent anode. As shown in Fig. 2a, the structure of the perovskite EL fibre was further characterized by a FIB-SEM system. The as-prepared perovskite fibre consists of ~150 nm PEDOT:PSS and PTAA as the HTL, a 65 nm thick QD layer (estimated by the thickness of the hybrid QD film) as the emitting layer, 50 nm TPBi as the ETL, and 3 nm Liq and 100 nm Al as the cathode. For a fibre-shaped device, the sheet resistance of the anode seriously affects the luminance uniformity. Herein, a multi-dip-coating process is adopted for anode fabrication, and the square resistance decreases with increasing dip-coating steps (Supplementary Fig. S5). The as-prepared PEDOT:PSS film is compact (Supplementary Fig. S6) and has a surface roughness of 1.57 nm (Supplementary Fig. S7). Due to the appropriate capillary force in the film-forming stage, uniform and bright electroluminescence can be easily realised. The current density-voltage-luminance curves of the perovskite EL fibres are shown in Fig. 2c. The QLEF still shows a considerable luminance of ~100 cd m^−2^ at 7 V after being bent dozens of times (Fig. 2c), and the corresponding current efficiency is 1.67 cd A^−1^ (Supplementary Fig. S8). It is well known that perovskite QDs are outstanding luminance materials for next-generation high-definition displays due to their high defect tolerance, quantum efficiency and narrow FWHM. By applying perovskite QDs as emitting materials in the electroluminescent fibre, the green electroluminescence perovskite fibre presents a narrow FWHM of 19 nm with chromaticity coordinates of (0.09,0.76) (Fig. 2d), which is the narrowest FWHM among all the reported fibre-shaped electroluminescent devices (Supplementary Table S1). As shown in the insert of Fig. 2d, the operating electroluminescent perovskite fibre shows uniform and bright green emission at 7 V. The QLEF fibres can also be easily woven into an ‘N’ pattern (Supplementary Fig. S9), showing great capability for wearable displays. Red fibres with uniform electroluminescence are also achieved by using the same composite strategy and exhibit chromaticity coordinates of (0.65,0.27) (Fig. 2e). The electroluminescent perovskite fibre can also be being bent to a bending radius of 4.5 mm during operation (Fig. 2f). In addition, the perovskite fibres can be wrapped around a pencil (Supplementary Fig. S10) and exhibit the potential to be used as decoration. The resulting fibres can also be woven into a glove and a sweater (Supplementary Figs. S11 and S12). When used as a fibre-shaped electroluminescent device, inherent merits, such as a wide viewing angle and no extra absorbing losses, also exist for the proposed perovskite, as shown in Supplementary Fig. S13.

### Photodetection properties of the perovskite fibres

**Fig. 3:**
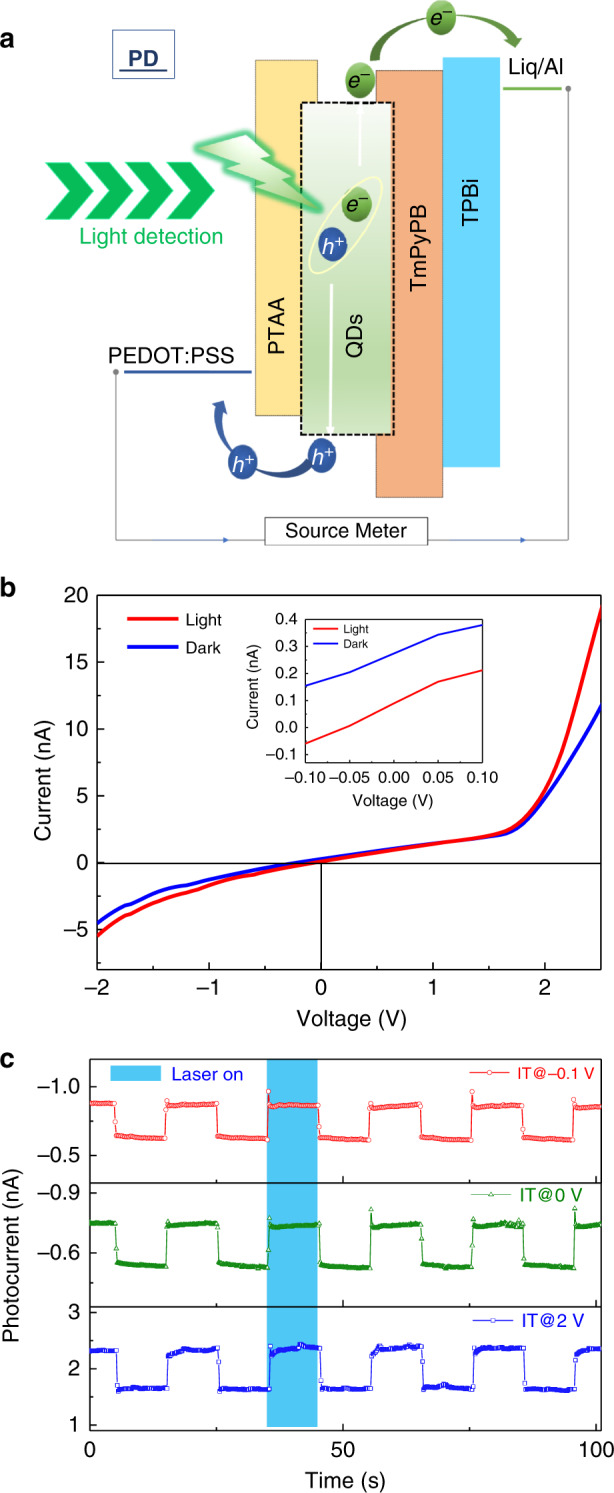
Photodetection function of the perovskite fibres. **a** Schematic illustration of the working mechanism (PD mode) of the light-emitting/detecting bifunctional perovskite fibre. **b** Photocurrent and dark current of the perovskite fibre. **c** I-T curves of the perovskite fibre under different bias conditions.

Apart from the fine electroluminescence quality of the perovskite fibres, their photoresponse capability was surprising. The energy band diagram of the proposed perovskite electroluminescent fibre is proposed in Fig. 3a, indicating that the fibre is capable of photodetection. As shown in Fig. 3b, a photocurrent arises from direct green laser (515 nm, 6.83 mW) irradiation in single detection mode at a rather small bias. The current varies significantly from dark to light conditions between −2 V and 2.5 V biases (Fig. 3b). In the insert of Fig. 3b, the *I–V* plot around zero bias is amplified, which shows that the QLEF can be self-powered, with a photocurrent response at zero bias. The self-powered property can be attributed to the band curvature in the space charge region induced by the Fermi level difference between the perovskite and the surrounding materials. The accompanying built-in field drives the charge separation process and gives rise to the photocurrent at zero bias. The *I-T* curves of the perovskite fibre under different bias conditions are shown in Fig. 3c. The *I*_light_*/I*_dark_ ratio of the QLEF is estimated to be ~1.5 under zero bias. The responsivity of the QLEF is calculated to be 1.86 × 10^−3^ mA W^−1^ at a −2 V bias and 3.15 × 10^−3^ mA W^−1^ at a 2.5 V bias under 515 nm illumination. The corresponding external quantum efficiency (EQE) of the QLEF in PD mode is calculated to be 0.45 × 10^−3^ % and 0.76 × 10^−3^%, respectively. Such a mediocre performance might result from the insufficient thickness of the active layer and could be further optimised by increasing the light capture, optimising the device structure, and improving the film quality of the active layer and contact between interfaces. In addition, the perovskite fibre has a photoresponse ability under illumination by 10 kHz square-wave modulated LD signals at a 0 V bias (Supplementary Fig. S14). Interestingly, the current also varies significantly at a forward bias of approximately 2 V, which shows potential for the construction of light-emitting/detecting bifunctional devices.

### Full-duplex light communication based on the EL-PD bifunctional fibres

**Fig. 4:**
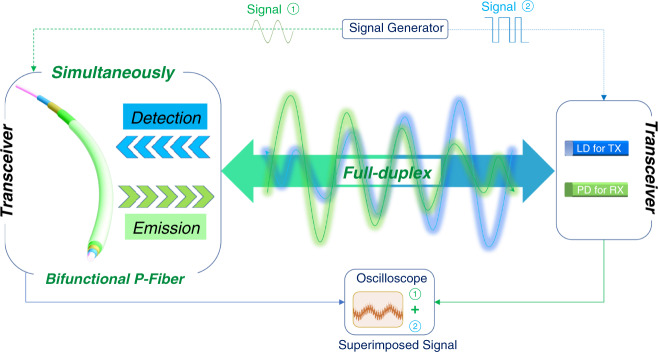
Demonstration of a wearable LiFi system based on the perovskite bifunctional fibres. **a** Schematic illustration of the full-duplex fibre-based wearable LiFi system based on the light-emitting/detecting bifunctional perovskite fibre. The transceiver on the left represents the mobile wearable terminal (MWT) based on the perovskite bifunctional fibres; the other transceiver represents the terminal that the MWT interacts with, which consists of a commercial photodiode (PD) and a laser diode (LD) as the receiver (RX) and transmitter (TX), respectively. Two sets of signals are used to test the capability of simultaneously transmitting and receiving information. Signal 1 (sine wave) is applied on the fibre to transmit data; Signal 2 (square wave) is applied on the LD to provide the incoming data that the fibre needs to identify. A superimposed signal that contains the information of the abovementioned two sets of signals is acquired by an oscilloscope, which is clearly distinguishable.

For a light communication system, bilateral real-time data transmission between the transmitting terminal and receiving terminal is one of the most critical issues. For wearable displays consisting of fabrics, a wide viewing angle of light-emitting fibres could satisfy the demand for multiangle data transmission. However, there are hardly any demonstrations of simultaneous data acquisition and data transmission through a single fibre. Therefore, a bifunctional fibre with both light emitting and photoresponse abilities is essential for bilateral real-time data transport and is the foundation of wearable fibre-to-fibre communication. Figure 4a demonstrates a wearable LiFi system based on the perovskite bifunctional fibres. Clearly, there are two transceivers in the system: the transceiver on the left represents the mobile wearable terminal (MWT) based on the perovskite bifunctional fibres; the other transceiver represents the terminal that the MWT interacts with, which consists of a commercial photodiode and a laser as a signal receiver (RX) and a transmitter (TX), respectively. A signal generator is used to generate different waveforms (first set of signals: voltage signal applied on the fibre; second set of signals: voltage signal applied on the commercial laser that induces the photoresponse of the fibre) to distinguish the detected signal and transmitted signals of the bifunctional fibres.

**Fig. 5:**
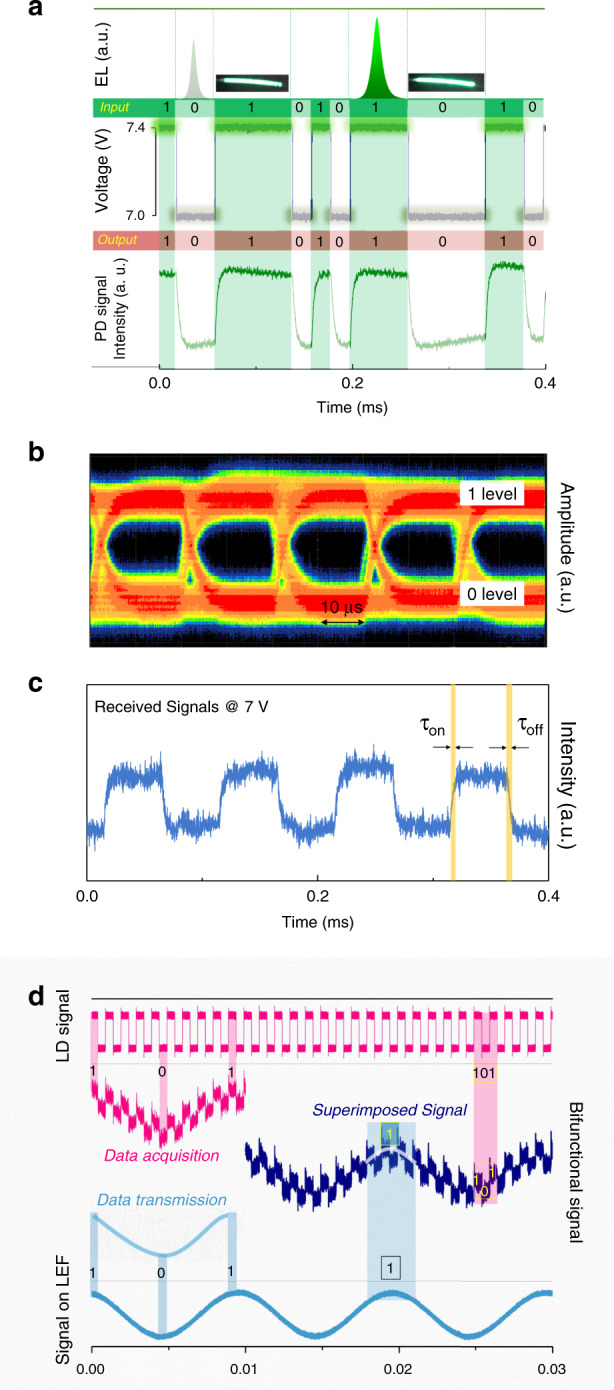
Full-duplex characteristics of the bifunctional fibre LiFi. **a** Illustration of the light intensity-related data transmission process using the perovskite fibre as the light source. Top, EL spectrum and images of the modulated EL fibre under 7 V and 7.4 V; middle, voltage signals applied on the fibre; and bottom, modulated fibre luminous signals acquired by a photodiode module. **b** Eye diagram versus data rate at 50 kb s^−1^ showing a clear open eye. **c** Square-wave signals received from the perovskite fibre under illumination by a 10 kHz LD. **d** Superimposed signals of the light-emitting/detecting bifunctional perovskite fibre when operating as a PD and an LED simultaneously.

Based on the above demands for a wearable LiFi system, we investigated the full-duplex LiFi properties of the as-prepared bifunctional fibres. The capability for data transmission was first investigated. The perovskite fibre functions as the transmitter and is directly driven by the alternating applied voltage generated by an arbitrary waveform generator. As shown in the middle of Fig. 5a, a peak-to-peak voltage of 400 mV and offset voltage of 7.2 V PRBS signal is adopted to modulate the voltage-related light-emission intensity, which contains data information. As shown at the top of Fig. 5a, the fibre shows different EL intensities and brightnesses under the modulated voltage signal. The modulated light containing data information is then received by the photodetector of the transceiver that the MWT interacts with. Afterwards, the photocurrent signals are amplified and exported to the oscilloscope (the bottom of Fig. 5a). If we assess the brighter light signals as the ‘1’ level and less bright light signals as the ‘0’ level, then we can clearly distinguish the 0 and 1 from the signal received by the photodetector that the transceiver MWT interacts with.

The corresponding eye diagram of the 50 kbit s^−1^ data rate clearly shows an open eye (Fig. 4c), which demonstrates the ability to transfer audio signals. An audio communication system can be easily constructed through acoustoelectric and photoelectric signal transformation with the help of external circuits (Supplementary Fig. S15)^23^. However, it is difficult for the unencapsulated fibre to stick to the whole audio transfer process. Despite the mediocre data rate that one QLEF has, the integration of fibres in wearable fabric will remarkably boost the data rate, which shows that QLEFs and fabrics based on them are very suitable platforms for data transmission.

Another necessary characteristic for the bifunctional fibre used in a wearable LiFi system is the photoresponse capacity at a forward bias that can drive EL emission, which was subsequently evaluated. Figure 5c shows transient photoresponse signals generated by the perovskite fibre at a forward bias of 7 V when the QLEF was irradiated by a 405 nm laser pulse driven at a 10 kHz frequency. The response and recovery processes are distinct and distinguishable. In addition, the response and recovery times are estimated to be 5 and 6 μs (defined as the time interval for the values to reach 90% and decrease to 10 % of the maximum amplitude, respectively). Notably, the response time and recovery times are comparable to those of outstanding fibre-shaped perovskite photodetectors and are also sufficient for text and audio transmission in optical communication systems^39,40^.

As illustrated in Fig. 4a, we further use two sets of signals to demonstrate the simultaneously occurring data transmission and acquisition processes. First, the 405 nm LD driven by the square wave signal is used to irradiate the QLEF (top of Fig. 5d), and the 100 Hz sine wave signal is applied on the QLEF to simulate the data transmission signal (bottom of Fig. 5d). As shown in the middle of Fig. 5d, the superimposed signal consists of two signals: the detected signal (data acquisition) and the transmitted signal (data transmission) of the fibre-based MWT. The superimposed signals can be extracted through a self-interference cancellation method. The simple binary data ‘1’ and ‘0’ are distinct, and data segments are well restored in the superimposed signal. The successful demonstration of bifunctional fibres verified the feasibility of a wearable full-duplex LiFi system based on EL-PD bifunctional perovskite fibres. Furthermore, both the electroluminescence and photodetection performance should be improved to realize bilateral real-time data transport between two fibres that belong to different interoperable LiFi terminals. In addition, different functions, such as biosensing, force sensing or other functions that are integrated into a single fibre or textiles, are also promising for future smart wearables.

In summary, we demonstrate wearable and colour tuneable perovskite light-emitting/detecting bifunctional fibres based on a hybrid strategy. The hybrid perovskite ink endows fibres with super-smooth QD films. This, combined with the small exciton binding energy and high carrier mobility of the perovskite QDs, enables the successful integration of electroluminescence and photodetection into a single fibre. The bifunctional fibres possess the narrowest luminescence spectrum of ~19 nm and, more importantly, the capability for simultaneously transmitting and receiving information. The successful fabrication of narrow emission full-duplex LiFi fibres paves the way for the fabrication and integration of low crosstalk interoperable smart wearables.

## Materials and methods

### Synthesis of perovskite NCs

The synthesis of perovskite NCs follows the reported room-temperature methods^28,41^. In short, caesium and formamidine acetate precursors were added to Pb^2+^ solution, followed by 2 min of stirring at room temperature in air. DDAB solution was added and reacted for 3 min, and ZnBr_2_ and TOAB were added to the crude solution and reacted for 5 min under continuous stirring. Ethyl acetate was used for QD purification. The precipitate was collected and redispersed in toluene, followed by the addition of poly(bis(4-phenyl)(2,4,6-trimethylphenyl) amine) (PTAA) and 1,3,5-tri[(3-pyridyl)-phen-3-yl]benzene (TmPyPB) (Xi’an Polymer Light Technology Corp, PLT).

### Device fabrication

PET fibres were sequentially cleaned with acetone and distilled water by ultrasonication. A PEDOT:PSS solution (Clevios PH 1000) was mixed with 5 wt % DMSO to improve the conductivity. Precleaned PET fibres were dip-coated in the PEDOT:PSS solution 9 times at a withdrawal speed of 60 mm min^−1^ to obtain the best conductivity and then baked for 30 min at 110 °C. A chlorobenzene solution consisting of PTAA was dip-coated on the fibres at a withdrawal speed of 120 mm min^−1^ and then baked for 20 min at 120 °C. Perovskite QD inks were dip-coated on the fibres at a withdrawal speed of 120 mm min^−1^ 3 times and then baked for 15 min at 60 °C. All dip-coating processes were performed in open air. Liq/Al (3 nm/100 nm) were deposited by thermal evaporation under a high vacuum of 2 × 10^−4^ Pa. The device active area was defined by the overlapping area between the PEDOT:PSS and Al electrodes.

### Characterization and device measurements

Cross sections of the perovskite fibre were characterized by a Zeiss Auriga FIB-SEM system. The UV-Vis spectra of the perovskite QD solutions were measured by a Shimadzu UV-3600 UV/VIS/NIR spectrophotometer. SEM images were obtained by using a FEI Quanta 250FEG system, and AFM images were acquired by a Bruker Multimode 8 AFM system. The PL spectra of the QDs were measured by using an Agilent Cary Eclipse spectrometer. The photodetector characteristics were measured by a Keysight B1500A semiconductor analyser. The EL spectra and *L*-*J*-V characteristics were collected by using a Keithley 2400 source metre, a spectroradiometer (Cs-2000, Konica Minolta), and a close-up lens at room temperature. The viscosities of the pure and molecule-doped QD solutions were acquired on a Malvern Rheometer Instrument (Kinexus Lab+, UK). The surface tensions of the pure and molecule-doped QD solutions were measured using a Kibron EZ Pi-Plus tensiometer. The light communication system consisted of an oscilloscope (DSO9254A, Keysight), a 405 nm OBIS laser, an arbitrary waveform generator (33622 A, KEYSIGHT) and a photodiode module (C12702-11, Hamamatsu).

## Supplementary information

SI

## Data Availability

All data needed to evaluate the conclusions in the paper are present in the paper and/or the Supplementary Information. Additional data related to this paper are available from the corresponding author on request.
