# Supplementary material for: Perovskite light-emitting/detecting bifunctional fibres for wearable LiFi communication
Source: Light Sci Appl. 2020 Sep 16;9:163. doi: 10.1038/s41377-020-00402-8 (PMC7494868; doi:10.1038/s41377-020-00402-8)
Supplement: Supplementary file 1 — SI [file 41377_2020_402_MOESM1_ESM.docx]

Supplementary Information for

**Perovskite Light-emitting/detecting Bifunctional Fibres for Wearable LiFi Communication**

Qingsong Shan^1#^, Changting Wei^1#^, Yan Jiang^2#^, Jizhong Song^1^*, Yousheng Zou^1^, Leimeng Xu^1^, Tao Fang^1^, Tiantian Wang^1^, Yuhui Dong^1^, Jiaxin Liu^1^, Boning Han^1^, Fengjuan Zhang^1^, Jiawei Chen^1^, Yongjin Wang^2^*, and Haibo Zeng^1^*

^1^ MIIT Key Laboratory of Advanced Display Materials and Devices, Institute of Optoelectronics & Nanomaterials, School of Materials Science and Engineering, Nanjing University of Science and Technology, Nanjing 210094, China

^2^ Peter Grünberg Research Center, Laboratory of Broadband Wireless Communication and Sensor Network Technology, Ministry of Education, Nanjing University of Posts and Telecommunications, Nanjing 210003, China

^#^ These authors contributed equally to this work.

*Authors to whom correspondence should be addressed: [songjizhong@njust.edu.cn](mailto:songjizhong@njust.edu.cn), [wangyj@njupt.edu.cn](mailto:wangyj@njupt.edu.cn), [zeng.haibo@njust.edu.cn](mailto:zeng.haibo@njust.edu.cn)

**Figure Legends**

**Supplementary Figure 1 |** SEM image of the dip-coated pure QD film.

**Supplementary Figure 2** | PL spectra of the pure and hybrid QD inks, respectively.

**Supplementary Figure 3** | (a) and (b), PL mapping of the dip-coated films based on pure QD and hybrid QD, respectively.

**Supplementary Figure 4** | (a) and (b), Surface tension of the pure and molecule doped QDs solution, respectively.

**Supplementary Figure 5 |** Sheet resistance of the dip-coated PEDOT:PSS film change with the number of dip-coating cycles.

**Supplementary Figure 6** | SEM image of the dip-coated PEDOT:PSS film.

**Supplementary Figure 7** | AFM image of the dip-coated PEDOT:PSS film.

**Supplementary Figure 8** | Current efficiency versus current density of the perovskite fibre.

**Supplementary Figure 9** | Photograph of the perovskite fibre being weaved to form the letter of “N”.

**Supplementary Figure 10** | Photograph of the perovskite fibres wrapped around a pencil.

**Supplementary Figure 11** | Photograph of the perovskite fibres woven into a glove.

**Supplementary Figure 12** | Image of the operating green emitting perovskite fibre being woven into a knitwear.

**Supplementary Figure 13** | Schematic illustration of the emission angle range of the perovskite electroluminescent fibre.

**Supplementary Figure 14** | Square-wave signals received by the perovskite fibre at 0 V bias under the illumination of a 10 kHz LD.

**Supplementary Figure 15** | Schematic of the audio communication system.

**Supplementary Table 1** | Viscosities of the pure and hybrid QD inks.

**
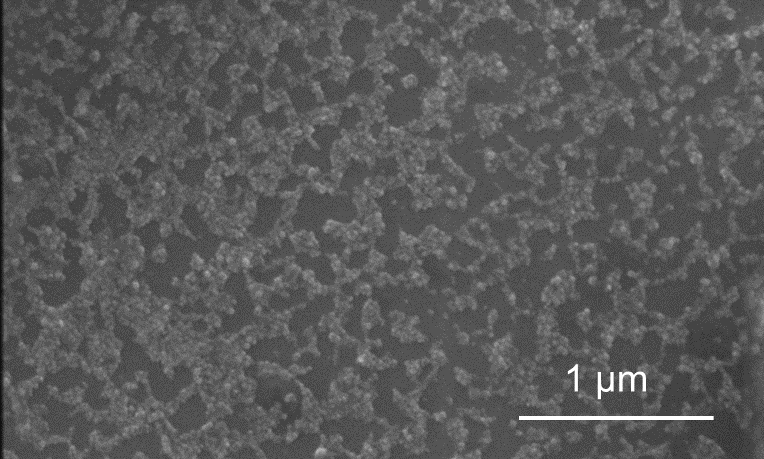
**

**Supplementary Figure 1 |** SEM image of the dip-coated pure QD film.





**Supplementary Figure 2** | PL spectra of the pure and hybrid QD inks, respectively.

**
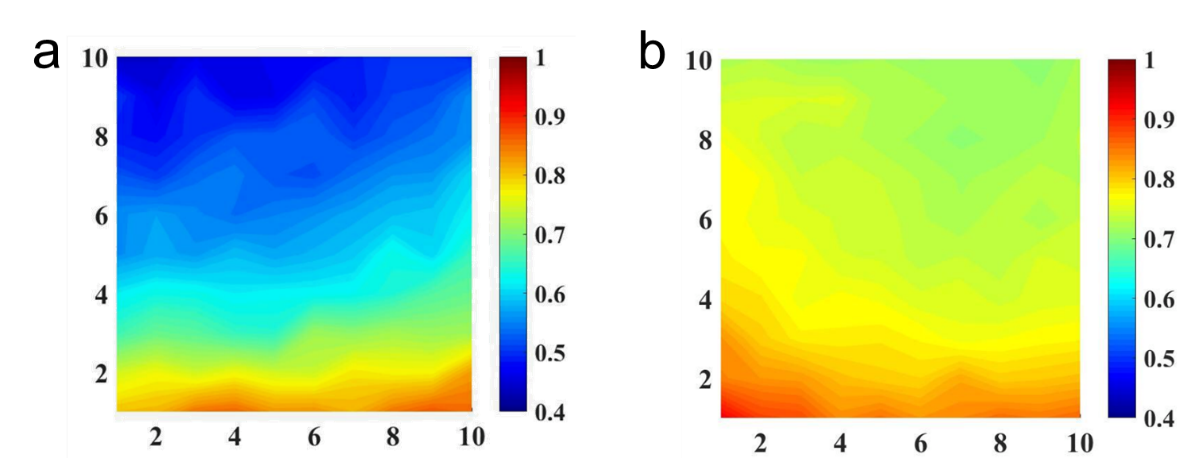
**

**Supplementary Figure 3** | (a) and (b) are PL mappings (10 μm ×10 μm) of dip-coated films based on pure inks and hybrid inks, respectively. The color scale represents the relative PL intensity.

**
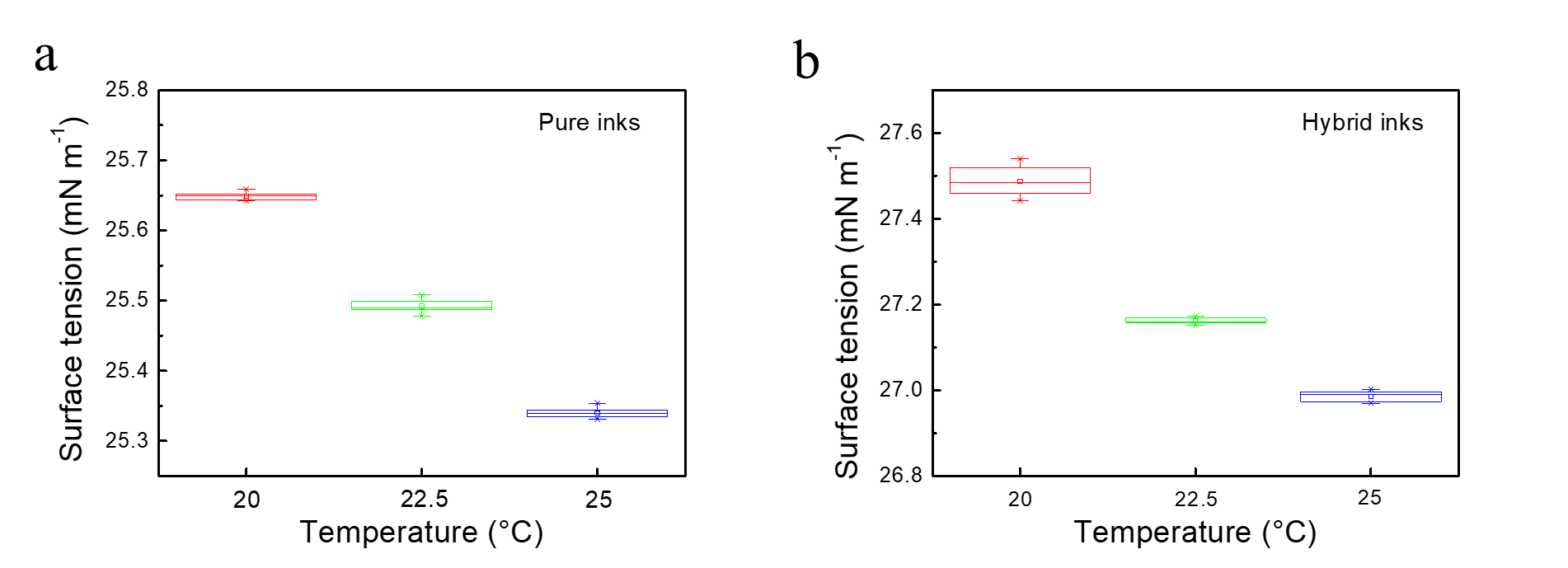
**

**Supplementary Figure 4** | (a) and (b), Surface tension of the pure and molecule doped QDs solution, respectively.


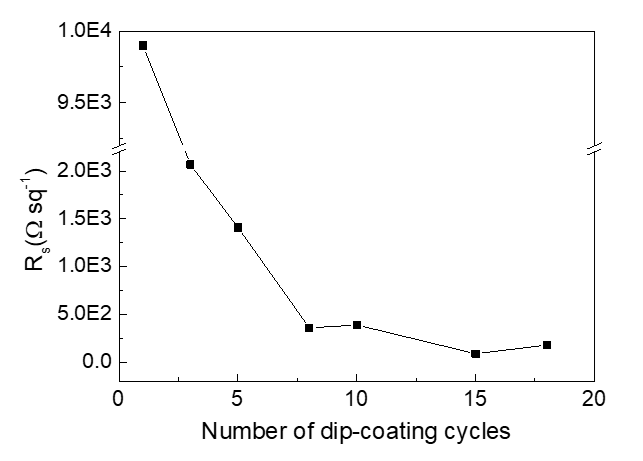


**Supplementary Figure 5 |** Sheet resistance of the dip-coated PEDOT:PSS film change with the number of dip-coating cycles.


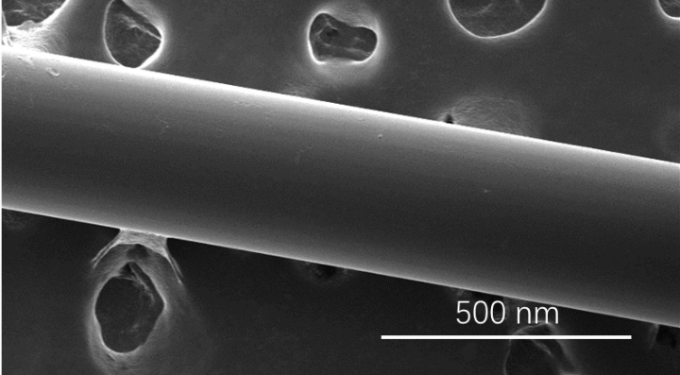


**Supplementary Figure 6** | SEM image of the dip-coated PEDOT:PSS film.


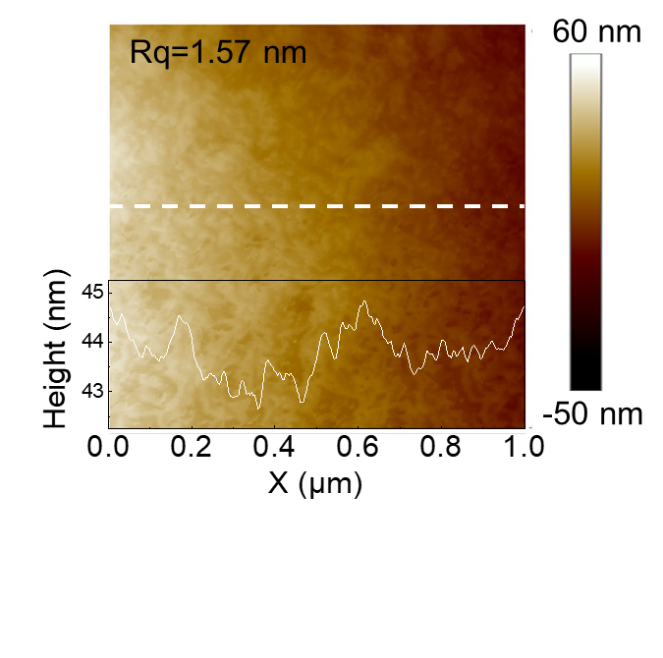


**Supplementary Figure 7** | AFM image of the dip-coated PEDOT:PSS film.


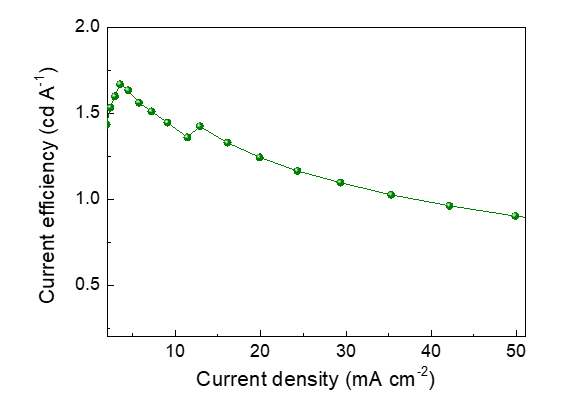


**Supplementary Figure 8** | Current efficiency versus current density of the perovskite fibre.


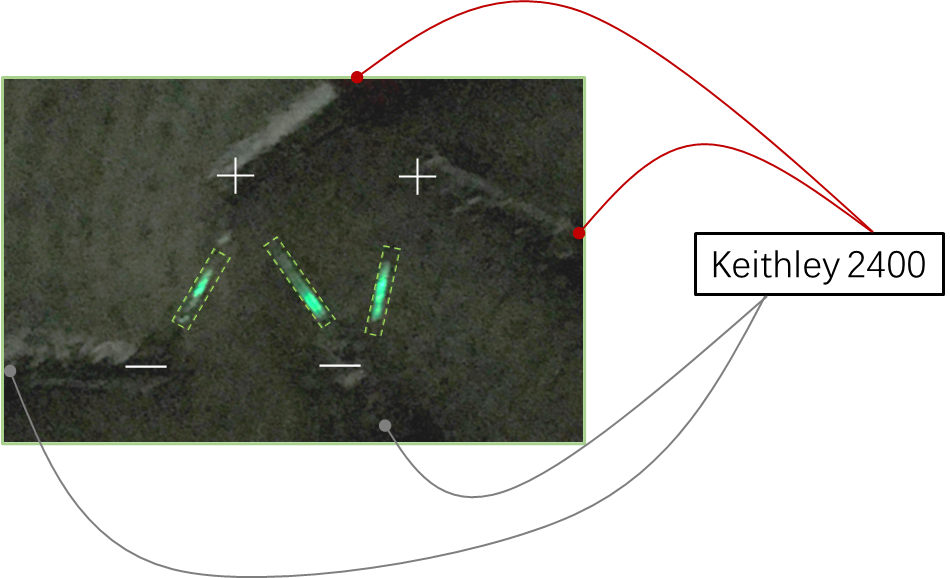


**Supplementary Figure 9** | Photograph of the perovskite fibre being weaved to form the letter of “N”.


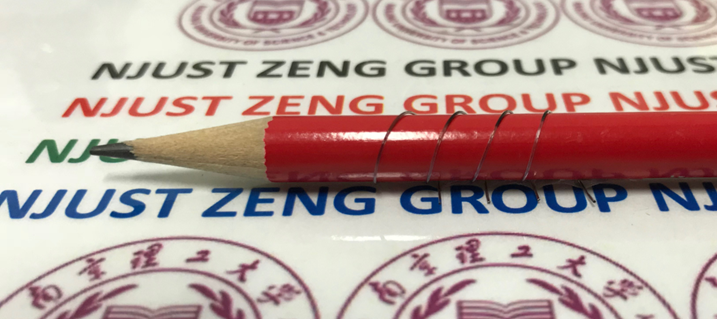


**Supplementary Figure 10** | Photograph of the perovskite fibres wrapped around a pencil.


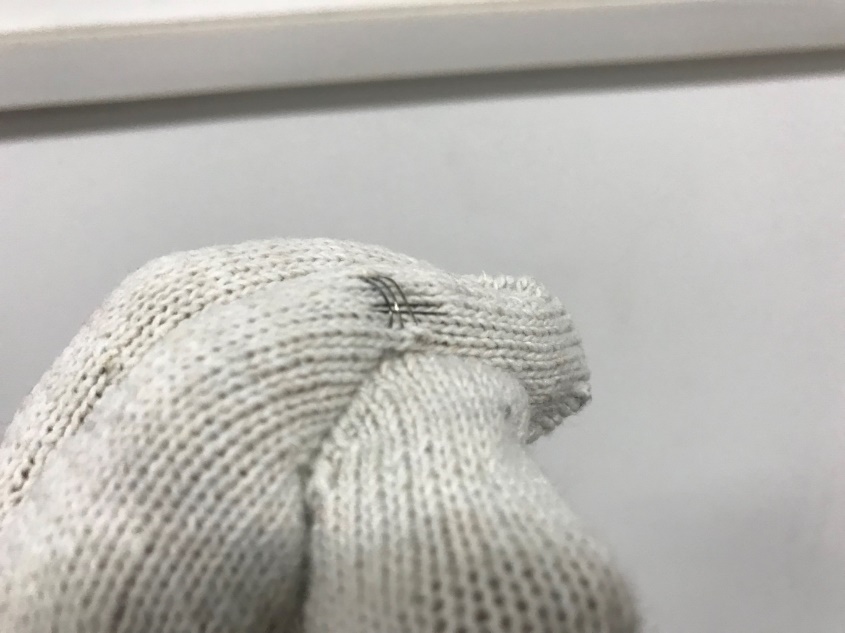


**Supplementary Figure 11** | Photograph of the perovskite fibres woven into a glove.


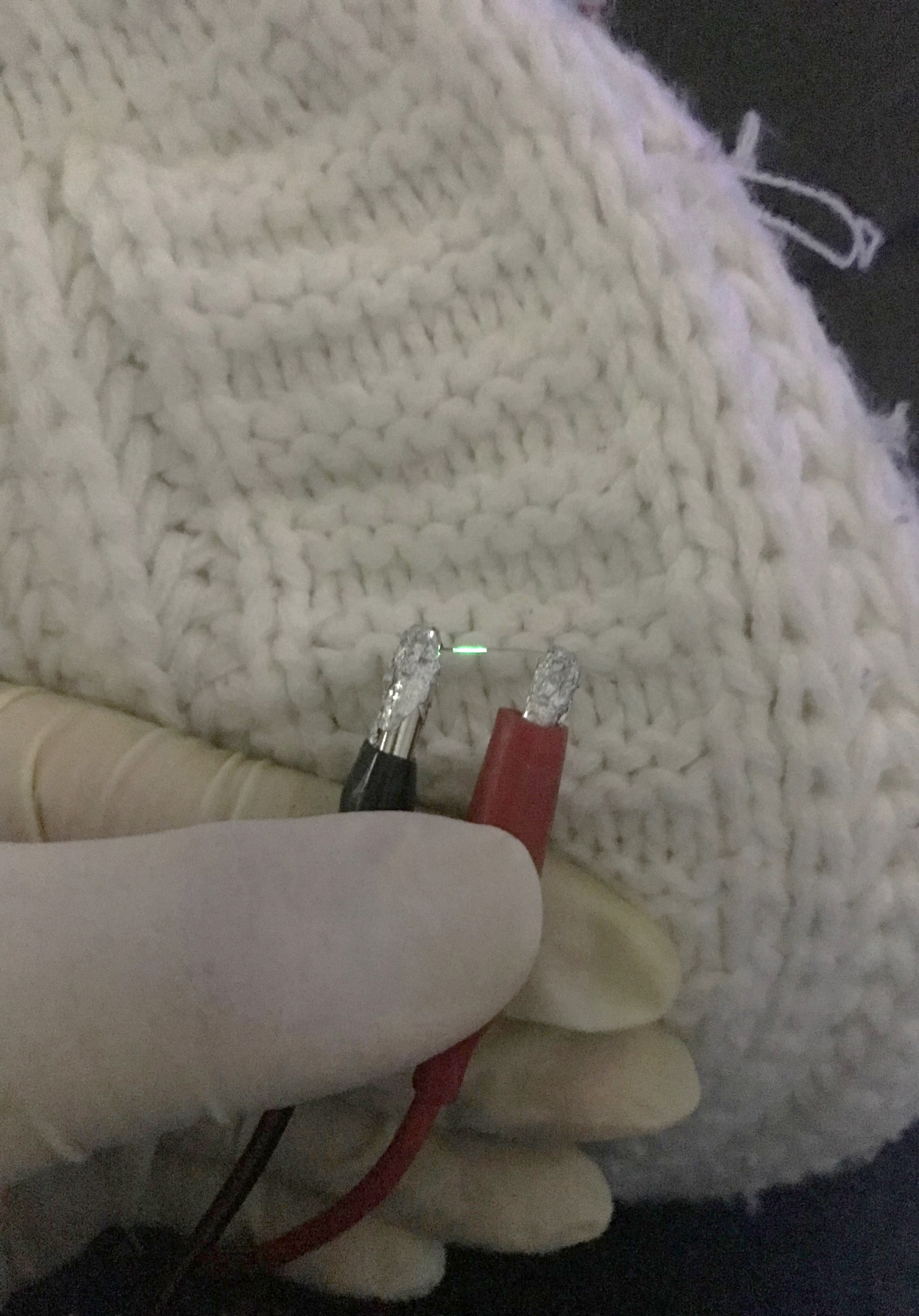


**Supplementary Figure 12** | Image of the operating green-emitting perovskite fibre being woven into a knitwear.


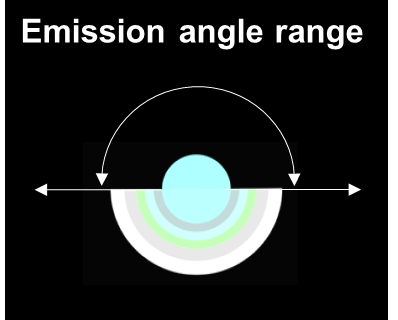


**Supplementary Figure 13** | Schematic illustration of the emission angle range of the perovskite electroluminescent fibre.





**Supplementary Figure 14** | Square-wave signals received by the perovskite fibre at 0 V bias under the illumination of a 10 kHz LD.


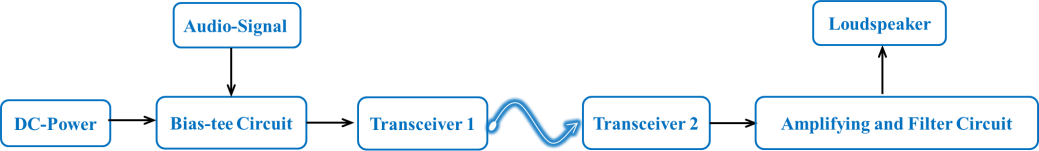


**Supplementary Fig. 15** | Schematic of the audio communication system.

The audio communication system are consist of a DC-power to drive transceiver for signal transmission, a bias-tee circuit to convert audio-signal into electric signal, a transceiver 1 to transmit data-contained light signal, a transceiver 2 to receive light signal and transmit the light signal into electric signal, an amplifying and filter circuit to transfer electric signal into audio signal.

**Supplementary Table 1** | Viscosities of the pure and hybrid QD inks.

| **Temperature(^o^C)** | **Viscosity (cp)-Pure inks** | **Viscosity (cp)-Hybrid inks** |
| --- | --- | --- |
| 20 | 0.457 | 0.88 |
| 22.5 | 0.453 | 0.727 |
| 25 | 0.448 | 0.696 |
